# Understanding Tick-Borne Encephalitis Virus Foci, a Tale of Two Mountains

**DOI:** 10.3390/pathogens12020265

**Published:** 2023-02-06

**Authors:** Alejandro Cabezas-Cruz, Pavle Banović

**Affiliations:** 1 ANSES, INRAE, Ecole Nationale Vétérinaire d’Alfort, UMR BIPAR, Laboratoire de Santé Animale, F-94700 Maisons-Alfort, France; 2 Clinic for Lyme Borreliosis and Other Tick-Borne Diseases, Department of Prevention of Rabies and Other Infectious Diseases, Pasteur Institute Novi Sad, 21000 Novi Sad, Serbia; 3 Department of Microbiology with Parasitology and Immunology, Faculty of Medicine, University of Novi Sad, 21000 Novi Sad, Serbia

What factors influence the formation and disappearance of tick-borne encephalitis virus (TBEV) foci? TBEV is member of the genus *Flavivirus*, family Flaviviridae, and is the most important viral pathogen transmitted by hard ticks in Europe [1]. In natural foci, this virus circulates between vertebrate hosts (e.g., small rodents of the genera *Apodemus* and *Myodes*) and the tick vectors that feed on them, mainly *Ixodes ricinus* but also *Dermacentor reticulatus* [2]. TBEV foci are complex microecosystems that are highly dependent on biotic and abiotic factors, which affect all components of TBEV transmission and the amplification chain [3]. The identification of TBEV foci is based on the detection of continuous virus transmission and virus dispersion from TBEV-positive ticks to rodents and other mammals [4]. Although tick vectors can become infected when feeding on viremic vertebrate hosts, one of the crucial elements for the formation and maintenance of TBEV foci is the occurrence of tick cofeeding, where the virus is disseminated from infected ticks to uninfected larvae or nymphs that are feeding simultaneously and in close proximity on the same nonviremic host [5,6].

One of the Editor’s Choice Articles in 2020 published in *Pathogens*, by Bournez et al. [6], showed that the dynamics of temperature oscillation in the period 2012–2018 influenced tick cofeeding in an examined area of the Vosgian forest, located in low mountains of the Alsace region in Eastern France. Their results suggest that cold temperatures in the mountain forest of Vosgian led to reduced larval activity in spring 2017 in territories with an altitude higher than 600 meters (m). This led to diachronicity in the questing activity of *Ixodes* sp. larvae and nymphs, decreasing the number of infected questing ticks and seropositive rodents, consequently leading to the disappearance of TBEV foci [6]. This was a focal event in the Vosgian mountain forest, since cases of human TBE were still occurring in the rest of the Guebwiller valley, which indirectly confirms the circulation of TBEV between its vectors and vertebrate reservoirs [6]. The study by Bournez et al. [6] adds evidence to the hypothesis that the focal distribution of TBEV is determined by the geographically variable degree of synchrony in the seasonal activity of larval and nymphal *I. ricinus* ticks [7].

The Vosgian forest encompasses a territory with complex microclimate characteristics that support various ecosystems, some of which are foci of different tick-borne pathogens (TBPs), including members of spotted fever group rickettsia (SFGR), *Borrelia burgdorferi* sensu lato complex, *Borrelia miyamotoi*, and *Anaplasma phagocytophilum* [8,9]. Similarly, the Fruška Gora mountain is a national park regarded as a suitable ecosystem for the vector *Ixodes ricinus* and transmitted TBPs such as *Borrelia burdgorderi* sensu lato complex, *B. miyamotoi*, and SFGR [10,11,12,13,14]. In addition to climatic conditions favorable for ticks, the presence of small rodents, such as yellow-necked mice (*Apodemus flavicollis)* and bank voles (*Myodes glareolus*) [15], may contribute to the circulation of TBPs, including TBEV, in the Fruška Gora mountain; however, although TBEV was previously detected in *I. ricinus* ticks from different sites of the Fruška Gora mountain [16], no locations in the mountain have been reported and/or regarded as TBEV foci [17]. Could similar climatic conditions drive the absence and/or disappearance of TBEV foci at the Fruška Gora mountain and the mountain forest in Vosgian? The Fruška Gora mountain is located in the Pannonian Plain (North Serbia), with an altitude of 539 m, and due to its height gradient and forest cover the climate has subcontinental characteristics. As the altitude increases the climate is more humid and colder, such that the ridges have the characteristics of a mountain climate with colder winters and fresher summers. There is thus a possibility that local climate factors occurring on the Fruška Gora mountain (e.g., cold winters and cold springs due to the presence of northern winds [18]) could reduce *Ixodes* sp. larval activity and therefore prevent the synchronic feeding of nymphs and larvae on small rodents, which may in turn decrease or eliminate the circulation of TBEV in established foci.

Given that Fruška Gora is a low mountain positioned in the Pannonian Plain, spanning across 266.7 km², there is a high chance that microclimatic factors favorable to the emergence of TBEV foci are present in some locations, such as lower areas where temperatures in spring are not highly affected by a cold climate. A similar hypothesis was proposed by Bournez et al. for an area surrounding the Vosgian forest, the Guebwiller valley, where TBE cases occur systematically [6]. A better understanding of the ecology of natural TBEV foci is essential for accurately predicting temporal variations in the risk of TBE for humans. Further studies on the temporal dynamics of natural TBEV foci in regions such as the Vosgian forest in France and the Fruška Gora mountain in Serbia offer an invaluable opportunity to empirically test how synchronous cofeeding between infected nymphs and uninfected larvae on small rodents favors TBEV transmission and increases the human risk of infection. For example, it could be interesting to compare the dynamics of the emergence and disappearance of TBEV foci in the Vosgian forest and Fruška Gora mountain and test whether these are stochastic or follow certain temporal patterns. The availability of TBEV-neutralizing antibody assays in Serbia since June 2022 [19] may enable the detection of autochthonous TBEV infection in the following years, while anamnestic data of TBE patients could reveal tick exposure localities of the Fruška Gora mountain and allow effective surveillance of TBEV hosts and vectors.

## References

[1] Amicizia D., Domnich A., Panatto D., Lai P.L., Cristina M.L., Avio U., Gasparini R. (2013). Epidemiology of Tick-Borne Encephalitis (TBE) in Europe and Its Prevention by Available Vaccines. Hum. Vaccin. Immunother..

[2] Ličková M., Havlíková S.F., Sláviková M., Slovák M., Drexler J.F., Klempa B. (2020). Dermacentor Reticulatus Is a Vector of Tick-Borne Encephalitis Virus. Ticks Tick Borne Dis..

[3] Borde J.P., Kaier K., Hehn P., Matzarakis A., Frey S., Bestehorn M., Dobler G., Chitimia-Dobler L. (2021). The Complex Interplay of Climate, TBEV Vector Dynamics and TBEV Infection Rates in Ticks—Monitoring a Natural TBEV Focus in Germany, 2009–2018. PLoS ONE.

[4] Dobler G., Hufert F., Pfeffer M., Essbauer S., Mehlhorn H. (2011). Tick-Borne Encephalitis: From Microfocus to Human Disease. Progress in Parasitology.

[5] Knap N., Avšič-Županc T. (2015). Factors Affecting the Ecology of Tick-Borne Encephalitis in Slovenia. Epidemiol. Infect..

[6] Bournez L., Umhang G., Moinet M., Boucher J.-M., Demerson J.-M., Caillot C., Legras L., Devillers E., Hansmann Y., Velay A. (2020). Disappearance of TBEV Circulation among Rodents in a Natural Focus in Alsace, Eastern France. Pathogens.

[7] Randolph S.E., Green R.M., Peacey M.F., Rogers D.J. (2000). Seasonal Synchrony: The Key to Tick-Borne Encephalitis Foci Identified by Satellite Data. Parasitology.

[8] Boyer P.H., Barthel C., Mohseni-Zadeh M., Talagrand-Reboul E., Frickert M., Jaulhac B., Boulanger N. (2022). Impact of Different Anthropogenic Environments on Ticks and Tick-Associated Pathogens in Alsace, a French Region Highly Endemic for Tick-Borne Diseases. Microorganisms.

[9] Richter D., Matuschka F.-R. (2006). Perpetuation of the Lyme Disease Spirochete Borrelia Lusitaniae by Lizards. Appl. Env. Microbiol..

[10] Potkonjak A., Kleinerman G., Gutiérrez R., Savić S., Vračar V., Nachum-Biala Y., Jurišić A., Rojas A., Petrović A., Ivanović I. (2016). Occurrence of Borrelia Burgdorferi Sensu Lato in Ixodes Ricinus Ticks with First Identification of Borrelia Miyamotoi in Vojvodina, Serbia. Vector Borne Zoonotic Dis..

[11] Banović P., Díaz-Sánchez A.A., Galon C., Simin V., Mijatović D., Obregón D., Moutailler S., Cabezas-Cruz A. (2020). Humans Infested with Ixodes Ricinus Are Exposed to a Diverse Array of Tick-Borne Pathogens in Serbia. Ticks Tick-Borne Dis..

[12] Banović P., Díaz-Sánchez A.A., Simin V., Foucault-Simonin A., Galon C., Wu-Chuang A., Mijatović D., Obregón D., Moutailler S., Cabezas-Cruz A. (2022). Clinical Aspects and Detection of Emerging Rickettsial Pathogens: A “One Health” Approach Study in Serbia, 2020. Front. Microbiol..

[13] Banović P., Díaz-Sánchez A.A., Galon C., Foucault-Simonin A., Simin V., Mijatović D., Papić L., Wu-Chuang A., Obregón D., Moutailler S. (2021). A One Health Approach to Study the Circulation of Tick-Borne Pathogens: A Preliminary Study. One Health.

[14] Banović P., Díaz-Sánchez A.A., Foucault-Simonin A., Mateos-Hernandez L., Wu-Chuang A., Galon C., Simin V., Mijatović D., Bogdan I., Corona-González B. (2023). Emerging Tick-Borne Spotted Fever Group Rickettsioses in the Balkans. Infect. Genet. Evol..

[15] Bjelic-Cabrilo O., Kostic D., Popovic E., Cirkovic M., Aleksic N., Lujic J. (2011). Helminth Fauna of the Bank Vole Myodes Glareolus (Rodentia, Arvicolinae) on the Territory of Fruska Gora Mountain (Serbia)–A Potential Source of Zoonoses. Bulg. J. Agric. Sci..

[16] Potkonjak A., Petrović T., Ristanović E., Lalić I., Vračar V., Savić S., Turkulov V., Čanak G., Milošević V., Vidanović D. (2017). Molecular Detection and Serological Evidence of Tick-Borne Encephalitis Virus in Serbia. Vector-Borne Zoonotic Dis..

[17] Kunze M., Banović P., Bogovič P., Briciu V., Čivljak R., Dobler G., Hristea A., Kerlik J., Kuivanen S., Kynčl J. (2022). Recommendations to Improve Tick-Borne Encephalitis Surveillance and Vaccine Uptake in Europe. Microorganisms.

[18] Stepanić A. (2011). Public Enterprise “National Park Fruška Gora”: Management Plan 2011–2022. https://www.npfruskagora.co.rs/data/dokumenti/program2011-2020.pdf.

[19] Dragonjić L.P., Vrbić M., Tasić A., Simin V., Bogdan I., Mijatović D., Cabezas-Cruz A., Banović P. (2022). Fatal Case of Imported Tick-Borne Encephalitis in South Serbia. Trop. Med. Infect. Dis..

